# Prediction of axillary lymph node metastasis in breast cancer patients based on ultrasonograhic-clinicopathologic features

**DOI:** 10.12669/pjms.41.1.10384

**Published:** 2025-01

**Authors:** Tuba Laiq, Zubia Masood, Hiba Siddiqui, Maliha Javed, M. Jawaid A. Mallick

**Affiliations:** 1 Tuba Laiq, MBBS Post graduate trainee, Department of Oncology, Dr. Ziauddin Hospital, Karachi, Pakistan; 2 Zubia Masood, FCPS, MHPE Associate Professor of Surgery Baqai Medical University, Consultant Surgeon, Dr. Ziauddin Hospital, Karachi, Pakistan; 3 Hiba Siddiqui, MBBS Post graduate trainee, Department of Oncology, Dr. Ziauddin Hospital, Karachi, Pakistan; 4 Maliha Javaid, MBBS Resident medical officer, Department of Oncology, Dr. Ziauddin Hospital, Karachi, Pakistan; 5 M. Jawaid A. Mallick, MD Consultant Oncologist, Head of Department of Oncology, Dr. Ziauddin Hospital, Karachi, Pakistan

**Keywords:** Axillary nodes, Breast cancer, Metastasis, Specificity, Sensitivity, Ultrasound

## Abstract

**Background & Objective:**

Determination of axillary lymph-node status plays a pivotal role in decision making for breast cancer treatment. Biopsy is the current standard of care but hold risks of complications as well. We aimed to find out the correlation of sonographic features of lymph node and histo-pathological findings, to predict axillary lymph-node metastasis in breast cancer patients.

**Method:**

This retrospective observational study included 176 breast cancer patients at a private tertiary care hospital from January 2019 to December 2023. The study calculated sensitivity, specificity and accuracy of ultrasound (US) in identifying ALN metastasis. Also, binary logistic regression analysis was used to demonstrate the association between suspicious findings on axillary US with pathology report. Patients who never had undergone axillary surgery or with insufficient data, were excluded from our study.

**Results:**

In our study Axillary US was found to be 84.2% sensitive, 48.1% specific, and 67.6% accurate in identifying nodal metastases. In this context, ALN metastases was strongly and independently correlated with cortical thickness > 3 mm and the absence of a fatty hilum (P <.05).

**Conclusion:**

Ultrasound was found to be highly sensitive but not specific in predicting metastatic lymph nodes in patients with breast cancer.

**Abbreviations:**
ALN: Axillary lymph node.
SLNBX: Sentinel lymph node biopsy.
ER: Estrogen receptor.
PR: Progesterone receptor.
HER-2: Human epidermal growth factor receptor 2
US: Ultrasound.
OR: Odds ratio.

## INTRODUCTION

Axillary Lymph node (ALN) metastasis is a well-known prognostic and predictive factor of survival among breast cancer patients. With 98.6% of five years overall survival rate in localized breast cancer, may reduce to 84.4% with diagnosis of nodal metastasis.^1^ Thus determination of the status of ALN metastasis is a crucial step in decision making for breast cancer treatment plan.^2^,^3^ The sentinel lymph node biopsy (SLNBX) is the standard of care and is highly sensitive for diagnosis but it is an invasive procedure that accompany risk of events like nerve injury, vascular damage, infection and lymphedema.^4^,^5^ This invasive surgical staging was questioned by the infamous ‘SOUND trial’ and results proved no benefit of any axillary surgery in early stage breast cancer.^3^ Thus, the guidelines no longer recommend SLNBX and axillary sampling in early stage hormone positive elderly breast cancer patients.^6^

The non-invasive, non-ionic and easily accessible US imaging of nodal morphology for determination of axillary metastasis is under study and various studies have been published to predict the nodal involvement on the basis of sonological data.^7^ A number of suspicion raising features have been discussed in previous studies that may foretell whether ALNs are malignant or not.^8^ Singh R et al. studied axillary ultrasound diagnostic accuracy and found it 61.5% sensitive and 75.6% specific.^9^ Similarly, another study found it 53.3% sensitive and 93.6% specific.^10^

In our region few studies have been done so far with contradictory results. Our study aims to identify clinical and sonographic features that points toward regional lymph node metastases.

## METHOD

This observational retrospective study comprised of 176 Breast cancer patients who underwent breast cancer surgery. It was designed to ascertain high risk US features that are predictors of ALN metastasis. For the aforementioned purpose complete data of diagnosed breast cancer patients from January 2019 to December 2023, were assembled with US features alongside with histo-pathological features data for correlation. Preoperative US, which was performed in all included patients, provided data on the extent of disease and suspicious features of lymphadenopathy. These characteristics which upraised suspicion of clinically involved nodes comprising of shape of LN, hyper echogenicity of LN, increased thickness of cortex of LN i.e. more than 3mm and absence of fatty hilum.

The pre-operative axillary ultrasound findings were compared with post-operative histopathology including sentinel lymph-node biopsy (SLNBX) and axillary lymph node dissection (ALND) data to conclude the precision of US diagnostic principles. All statistical analyses were performed using Microsoft Excel 2011 and SPSS software version 20. Using the logistic regression model, odds ratios with 95% confidence intervals were calculated. The comparison of pathologically positive nodes with each ultrasonography characteristic under study were considered significant if the p-value was less than 0.05. Also, data was calculated for positive and negative ratios in diagnosis of lymph nodes on US. Furthermore, sensitivity, specificity and accuracy along with Positive predictive value and negative predictive value of the axillary US in diagnosing nodal involvement were determined using the formulas for sensitivity, specificity, and accuracy (Table-IV).

### Ethical Approval:

This study was conducted at a tertiary care hospital after taking ethical committee approval dated July 11, 2023 (reference code: 7200523TLRAD).

The study included only Biopsy proven breast cancer patients with pre surgery ultrasound axillary lymph nodes, having detailed nodal sonological features, along with post-operative axillary lymph nodes histopathology having features reported in detail. Patients who did not underwent ALN dissection or those with no or limited pre-operative records were excluded from the study.

## RESULTS

The study evaluated a total of 176 breast cancer patient’s data and incorporated for analysis. The demographics and clinic-pathologic features of counted in patients are presented in Table-I. The studied reports with positive pathological nodes were found in 54% of all cases, where the studied pre-surgical US reports predicted 69.3% of patients to be having ALN metastasis. Our retrospective evaluation of axillary ultrasounds was focused on changes in lymph node’s shape, echogenicity, size of the cortex and integrity of hilum (Table-II), where the most frequent altered morphologic feature observed was shape in about 58% of the reports. However, it was statistically non-significant (p-value: .391, OR: .726 (0.349–1.511)) as well as echogenicity was found statistically non-significant (P-value .966, OR 1.024 (0.347–3.017)). However, absence of fatty hilum in 34.7% (P-value .013, OR .348 (1.52–0.797)) and increased cortical thickness more than 3mm in 46.0% (P-value .001, OR .265 (0.122–0.574) was statistically significant (Table-III).

**Table-I:** Patient’s Demographics and Clinico-pathological Features.

		Frequency	Percent
Age	18-30 Years	3	1.7
	31-40 Years	19	10.8
	41-50 Years	51	29.0
	51-60 Years	42	23.9
	61-70 Years	44	25.0
	71-80 Years	11	6.3
	80 and above	6	3.4
Menopausal Status	Premenopausal	45	25.6
	Peri menopausal	19	10.8
	Post-menopausal	112	63.6
Type of Cancer	Ductal Carcinoma	148	84.1
	Lobular Carcinoma	9	5.1
	Others	19	10.8
Grade of Cancer	Grade I	11	6.3
	Grade II	92	52.3
	Grade III	73	41.5
Receptor Status	ER-/PR-/Her2-	42	23.9
	ER=/PR=/Her2+	10	5.7
	ER+/PR+/Her2-	102	58.0
	ER-/PR-/Her2+	22	12.5
Ki-67 Proliferative Index	Low (<15%)	65	36.9
	High (>15%)	111	63.1

**Table-II:** Positive predictive values for individual axillary ultrasonography characteristics in detecting pathologically positive axillary lymph nodes.

Characteristics Features On Ultrasound (n=176)	N	TP	FP	PPV %
Irregular shape	102	66	36	62.2
Absence of fatty hilum	61	48	13	78.6
Increased echogenicity	28	21	07	75
Cortical thickening >3mm	81	62	19	76.5

FP = false positive, PPV = positive predictive value, TP = true positive. PPV = TP / (TP + FP).

**Table-III:** Binary logistic regression analysis predicting pathological lymph node involvement according to discrete ultrasound features.

Ultrasound Features (N=176)	n	Pathologically Positive Node, n (%)	P-Value	Or (95% ci)
Irregular Shape			.391	
Not Present	74	29 (39.1%)		1
Present	102	66 (64.7%)		.726 (0.349–1.511)
Increased Echogenicity			.966	
Not Present	148	74 (50%)		1
Present	28	21 (75%)		1.024 (0.347–3.017)
Increased Cortical Thickness			.001	
Not Present	95	33 (34.7%)		1
Present	81	62 (76.5%)		.265 (0.122–0.574)
Absence Of Fatty Hilum			.013	
Not Present	61	48 (78.6%)		1
Present	115	47 (40.8%)		.348 (1.52–0.797)

The calculated false negative ratio of lymph nodes that had no pathological appearance on US but were found positive on histopathology was 15.7% (15/15+80). Conversely false positive ratio was 51.8% (42/42+39), where abnormal looking lymph nodes on US were found negative on post-surgical histopathology. Thus, Sonological results were compared with the histo-pathological results. The output data’s accuracy, sensitivity, and specificity were computed. Its result showed that it has 48.1% specificity, 84.2% sensitivity, and 67.6% accuracy (Table-IV). Additionally, 65.5% was the estimated positive predictive value. However, the negative predictive value of US in diagnosis for metastatic lymphadenopathy was found to be 72.2% (Table-IV).

**Table-IV:** Sensitivity, Specificity and accuracy of Ultrasound.

		Axillary LN Positive on Histopathology		

		Negative	Positive	
Ultrasound	Negative	True negative TN= 39	False negative FN = 15	Negative predictive value = TN / (FN + TN) =39/54 =72.2%
	Positive	False positive FP =42	True positive TP = 80	Positive predictive value =TP / (TP + FP) =80/122 =65.5%
		Specificity = TN/(TN+ FP) 39/(39+42) =39/81 =48.1%	Sensitivity =TP / (TP + FN) =80/95 =84.2%	Accuracy = TP + TN /(TP + FP + TN + FN) =80+39/80+42+39+15 = 119/176 =67.6%

## DISCUSSION

Our study found that the ultrasound is 84.2% sensitive in diagnosing metastatic ALN in breast cancer patients which is consistent with the previously reported ranges of sensitivity of 49-87%.^11^ A study from East Asia region on diagnostic accuracy of Ultrasound also found ultrasound to be 88.24% sensitive and 94.16% specific.^12^ However, in our study, specificity was found lower than the frequent reported range in most of the studies (55-97%).^11^ This predictability to diagnose pathological nodes in those who have no active breast cancer could be the reason of reduced specificity as the chosen population in our study was already diagnosed cases of breast cancer.^13^ A similar study with diagnosed population found sensitivity 83.5% and specificity 51.5%, similar to our findings.^14^ Rukanskienė et al while discussing the sensitivity and specificity of pre-operative ultrasound found it 52.9% sensitive and 89.6% specific, but in presence of ≥ 3 positive nodes the sensitivity was raised to 72.2%. This study also states in presence of high nodal burden disease ultrasound accuracy and NPV can be as high as 88.7% and 98.3%, respectively.^15^ Furthermore, Javed N et al. also found axillary ultrasound’s NPV of 80.2%. The results also concluded that axillary ultrasound false negative ratio is significantly lowered in presence of high risk features including larger primary tumor size, higher grade and aggressive tumor biology.^16^ Thus, nodal positivity on preoperative axillary US have significantly higher nodal burden disease on post-operative histopathology.^17^ In our study pre-op data was deficient in marking the presence of nodal burden, thus any difference of sensitivity and accuracy on the basis of high vs. low burden disease could not be studied.

In subject of negative predictive value (NPV), positive predictive value (PPV) and accuracy, Singh et al.^9^ found negative predictive value of 68%, positive predictive value and accuracy of 69% of US which are comparable to our study.^10^ In contrast to a study by Abidi et al, where positive predictive value was 75% and negative predictive value was 65%. Our study found US has high negative predictive value means if there are no suspicious findings on US, there is high probability of the node being negative on histopathology and the patient may not need any axillary surgery.^18^ The wide range and variability among different studies could be due to the difference in level of expertise and the variable criteria for examining lymph node on US. Also, the combination of criteria did not have a satisfactory overall diagnostic performance, in contrast to previous investigations.^11^

Our analysis marked thickened cortex and absent fatty hilum have strong association to predict axillary metastasis. Similar to a predictive model that also found these features along with lymph node long axis, short axis, and longitudinal-transverse *ratio (L*/*T ratio) is greatly predictive of ALN involvement.^19^* Stachs A et. al shared alike model and correspondingly highlighted the increased probability of lymph nodes is associated with large tumor size.^20^ Likewise, Tandon et al. found large tumor size and higher tumor grade are predictors of high nodal burden disease.^17^ In our study majority of cases were grade II and III, thus comparable analysis on association of metastatic lymphnode per grade of tumors was not performed.

Even with multiple studies suggested nomograms and predictive models there stays a chance of missing nodal infiltration on imaging likely because of microscopic disease of ≤5mm in size is depicted by only 25% of cases.^20^ In early stage breast cancer, there stands a risk of missing nodal metastasis on US. Mushtaq et al found 17.2% of patients with clinically negative axilla and no positive findings on US, were found positive on histopathology.^21^ In our study where thickened cortex had significant association in diagnosing metastasis, 34.7% were found positive in the absence of variability in cortex of lymph node. These outcomes require further analysis on factors that cause nodal positivity in such patients. The ongoing INSEMA trial and Dutch BOOG 2013–08 trial results are awaited that aim to answer similar objective and safety of skipping SLNBX in early stage disease.^6^

The proper assessment of axilla status is crucial and in presence of high risk features including large tumor size, high Ki-67 (proliferative index) and high grade disease further assessment may be required for exclusion of probability of nodal metastasis which carry higher risks of nodal involvement.^22^ With high sensitivity in diagnosing axillary metastasis, US is a non-invasive modality and can predict nodal positivity in breast cancer patients. But since the US is operator dependent, only trained breast radiologist should perform the scan and correlation of radiological findings with disease biology is highly suggested.

### Limitations:

This is a retrospective, single-center study. The radiological data incorporated was inclusive of scans from different centers with unknown radiologist’s expertise.

## CONCLUSION

Axillary ultrasound in breast cancer patients is highly sensitive but not very specific in detecting ALN metastasis.

### Authors’ Contribution:

**TL, ZM, HS & MJ:** Substantial contributions to study design, analysis and interpretation of data, Manuscript writing, has given final approval of the version to be published.

**MJAM:** Critical review, overall supervision.

All authors are accountable for all aspects of the work in ensuring that questions related to the accuracy or integrity of any part of the work are appropriately investigated and resolved.
